# Mutant epidermal growth factor receptor enhances induction of vascular endothelial growth factor by hypoxia and insulin-like growth factor-1 via a PI3 kinase dependent pathway

**DOI:** 10.1054/bjoc.2001.1805

**Published:** 2001-05

**Authors:** K Clarke, K Smith, W J Gullick, A L Harris

**Affiliations:** Growth Factor Group, Molecular Oncology Laboratories, Imperial Cancer Research Fund, John Radcliffe Hospital, Oxford OX3 9DU, UK; Imperial Cancer Research Fund Oncology Unit, Hammersmith Hospital, London W12 0NN, UK

**Keywords:** angiogenesis, EGFR, VEGF

## Abstract

Over-expression of truncated epidermal growth factor receptor (EGFR) occurs in a variety of malignancies including glioblastoma multiforme, breast and lung cancer. The truncation deletes an extracellular domain and results in constitutive activation of the receptor. NIH3T3 cells were transfected with full length or truncated human EGFR and differences in growth rates in vivo and in vitro analysed. A growth advantage was seen for cells expressing mutant receptor compared to full length EGFR in vivo only. Administration of an anti-mutant EGFR antibody to mice transiently reduced the growth rates of mutant tumours, confirming that the mutant receptor itself was important in this enhanced tumorigenicity. This showed that stimuli present in vivo and not in vitro may be contributing to growth. We therefore analysed the regulation of the angiogenic factor vascular endothelial growth factor (VEGF). Although levels of secreted VEGF did not differ significantly between wild-type and mutant EGFR cell lines when grown in vitro under normoxic conditions, following exposure to 0.1% hypoxia levels of VEGF produced by mutant cells increased 3.5–6.6 fold compared to 2 or less for full length EGFR cells. The fold induction was influenced by experimental conditions, including cell confluence and percentage of fetal bovine serum, but was consistently higher for mutant cell lines. The increase in VEGF under hypoxic conditions was blocked by the addition of PI3 kinase inhibitors, indicating that the latter pathway is important in the hypoxic stress response. Basal levels were not affected. Addition of insulin-like growth factor-1 also increased levels of VEGF under normoxic conditions in the mutant cells and no further increase was seen when added to cells exposed to 0.1% oxygen, indicating that levels of VEGF were already maximally stimulated. These results show that the mutant EGFR interacts with other growth factors and hypoxia to regulate VEGF via a PI3 kinase pathway, and suggests a specific role for anti-mutant EGFR antibodies and PI3 kinase inhibitors as therapy of this specific tumour target. © 2001 Cancer Research Campaign www.bjcancer.com


*British Journal of Cancer* (2001) **84**(10), 1322–1329
© 2001 Cancer Research Campaign
doi: 10.1054/ bjoc.2001.1805, available online at http://www.idealibrary.com on IDEAL®

http://www.bjcancer.com

### Mutant epidermal growth factor receptor enhances induction of vascular endothelial growth factor by hypoxia and insulin-like growth factor-1 via a PI3 kinase dependent pathway

**K Clarke[1], K Smith[1], WJ Gullick[2] and AL Harris[1]**

[1]Growth Factor Group, Molecular Oncology Laboratories, Imperial Cancer Research Fund, John Radcliffe Hospital, Oxford OX3 9DU, UK; [2]Imperial Cancer Research Fund Oncology Unit, Hammersmith Hospital, London W12 0NN, UK

**Summary** Over-expression of truncated epidermal growth factor receptor (EGFR) occurs in a variety of malignancies including glioblastoma multiforme, breast and lung cancer. The truncation deletes an extracellular domain and results in constitutive activation of the receptor. NIH3T3 cells were transfected with full length or truncated human EGFR and differences in growth rates in vivo and in vitro analysed. A growth advantage was seen for cells expressing mutant receptor compared to full length EGFR in vivo only. Administration of an anti-mutant EGFR antibody to mice transiently reduced the growth rates of mutant tumours, confirming that the mutant receptor itself was important in this enhanced tumorigenicity. This showed that stimuli present in vivo and not in vitro may be contributing to growth. We therefore analysed the regulation of the angiogenic factor vascular endothelial growth factor (VEGF). Although levels of secreted VEGF did not differ significantly between wild-type and mutant EGFR cell lines when grown in vitro under normoxic conditions, following exposure to 0.1% hypoxia levels of VEGF produced by mutant cells increased 3.5–6.6 fold compared to 2 or less for full length EGFR cells. The fold induction was influenced by experimental conditions, including cell confluence and percentage of fetal bovine serum, but was consistently higher for mutant cell lines. The increase in VEGF under hypoxic conditions was blocked by the addition of PI3 kinase inhibitors, indicating that the latter pathway is important in the hypoxic stress response. Basal levels were not affected. Addition of insulin-like growth factor-1 also increased levels of VEGF under normoxic conditions in the mutant cells and no further increase was seen when added to cells exposed to 0.1% oxygen, indicating that levels of VEGF were already maximally stimulated. These results show that the mutant EGFR interacts with other growth factors and hypoxia to regulate VEGF via a PI3 kinase pathway, and suggests a specific role for anti-mutant EGFR antibodies and PI3 kinase inhibitors as therapy of this specific tumour target. © 2001 Cancer Research Campaign http://www.bjcancer.com

**Keywords**: angiogenesis; EGFR; VEGF

Over-expression of the tyrosine kinase proto-oncogenic receptor epidermal growth factor receptor (EGFR), has been reported to occur in many malignancies including gliomas, head and neck tumours, lung, breast and bladder cancer (Gullick, 1991). It is associated with a poor prognosis in several tumour types including breast cancer (Harris et al, 1992; Salomon et al, 1995). Rearrangements in the *EGFR* gene have been reported in cases where EGFR is amplified. One mutant, EGFR type III (EGFRvIII), in which there is a deletion of 267 amino acids in the receptor's extracellular domain, occurs during malignant progression (Ekstrand et al, 1992). It is similar to EGFR in its ability to dimerise and autophosphorylate but is constitutively active and does not bind EGF (Ekstrand et al, 1994; Schmidt et al, 1998). EGFRvIII has been observed in 25–60% of gliomas, depending on the method of screening (Wong et al, 1992; Wikstrand et al, 1995), and a significant percentage of non-small cell lung (16%) and breast cancers (27–78%) (Moscatello et al, 1995; Wikstrand et al, 1995) but has not been detected in normal human tissue (Wikstrand et al, 1995).

*Received 27 June 2000*
*Revised 22 February 2001*
*Accepted 28 February 2001*

*Correspondence to:* AL Harris

Previous work has indicated a link between EGFR expression and angiogenesis (Petit et al, 1997). Angiogenesis is a complex process regulated by stimulatory and inhibitory factors, which result in the formation of new blood vessels from pre-existing endothelium. It is essential for progression of tumours beyond several millimetres in diameter. (Folkman, 1990). EGFR expression in tumour sections has been shown to have a positive correlation with hot spot microvessel density, a measure of angiogenesis (De Jong et al, 1998). Vascular endothelial growth factor (VEGF) is a potent regulator of angiogenesis, acting as a specific mitogen for endothelial cells, and as such has a major role in tumour-induced angiogenesis (Fidler and Ellis, 1994). Following treatment of cells over-expressing EGFR in well oxygenated conditions with an anti-EGFR antibody, C225, down-regulation of VEGF production has been described, suggesting a role for EGFR as an inducer of VEGF (Petit et al, 1997). In vivo administration of the same antibody to mice with established epidermoid cancers resulted in reduced growth and down-regulation of VEGF (Petit et al, 1997).

Environmental stimuli are known to regulate VEGF. Of these, hypoxia is one of the best characterized and can transiently upregulate VEGF (Shweiki et al, 1992). Increased VEGF production has been documented in cells over-expressing mutant ErbB2/neu (another EGFR-like oncogenic tyrosine kinase) compared to untransformed parental cell lines under resting conditions, and is further increased by conditions that mimic hypoxia ($CoCl_2$ 100 μM) (10). In vitro, VEGF expression has been shown to increase within 3–6 hours of hypoxic exposure (Levy et al, 1995). Hypoxia increases VEGF expression acutely through increase in transcription and prolongation of mRNA half life (Levy et al, 1995). Cytokines and growth factors are also known to mediate VEGF expression (Akagi et al, 1998). However, not all factors increase expression in all tumour systems and response to known stimulators, including hypoxia, varies between cell lines and with experimental conditions (Scott et al, 1998).

The signalling pathway by which hypoxia increases VEGF, has not been fully defined. Tyrosine phosphorylation is a key element in the signal transduction mediated by EGFR. A number of proteins are activated by EGFR including ras, which plays a pivotal role in relaying signals from the cell surface to a cascade of intracellular serine threonine kinases, resulting in the activation by phosphorylation of various transcriptional factors (Rak et al, 1995; Whitmarsh and Davis, 1996). Whilst ras appears to be important in the regulation of VEGF (Feldkamp et al, 1999), other oncogenes, tumour suppressor genes and growth factors must also play a role as evidenced by the finding that virtually all colon cancer cells express VEGF, yet only 50% have a mutant k-ras allele (Rak et al, 1995). Phosphatidylinositol-3-kinase (PI3 kinase), which is constitutively active in EGFRvIII transformed cells (Moscatello et al, 1998), also appears to be important. PI3 kinase plays an important role in many cellular processes including mitogenesis and cell survival. Production of PI3 kinase activates protein kinase B (PKB)/Akt. The survival signal mediated by various growth factors and cytokines may be dependent on the PI3 kinase/PKB signal transduction pathway (Gerber et al, 1998).

Because of differences in effect of mutant EGFRvIII on in vivo and in vitro growth (Nishikawa et al, 1994), we have used NIH3T3 cells transfected with full length or mutated EGFRvIII, to investigate modulation of VEGF production by environmental stimuli. NIH3T3 cells were chosen based on their known low expression of VEGF and low levels of activated Ras (Guha et al, 1997) thereby allowing the effects of mutant EGFR on the expression of VEGF to be investigated on a low Ras background, without multiple other pathways and thus to elucidate the role of the mutant. The role of PI3 kinase and its down-stream target Akt3 were investigated using specific PI3 kinase inhibitors. The role of growth factors in increasing VEGF was also analysed using EGF, and IGF-1, a known stimulator of VEGF in other tumour systems.

### MATERIALS AND METHODS

### Materials

Recombinant human insulin-like growth factor-1 (IGF-1) was purchased from Life Technologies (GIBCO BRL, Paisley, UK), and epidermal growth factor (EGF) and PI3 kinase inhibitors LY 294002 and wortmannin, from Sigma (Louis, MO).

### Antibodies

EGFR1, a mouse monoclonal antibody which interacts only with full length human EGFR (Waterfield et al, 1982), and DH8.3 an $IgG_1$ antibody which recognizes mutant but not full length EGFR (Hills et al, 1995), were obtained from ICRF research monoclonal antibody facility (Clare Hall, Lincoln's Inn Fields, London, UK).

### Cell lines

The generation of stably transfected NIH3T3 cell lines, DH-EGFR-E (which expresses full length EGFR cDNA), DH-EΔ801-P (expresses truncated receptor), and DH-L-12 (empty vector), have been previously described (Hills et al, 1995).

Cell lines were maintained in Dulbecco's modified Eagles medium (DMEM; Clare Hall Laboratories, Imperial Cancer Research Fund, South Mimms, UK), supplemented by 10% heat-inactivated fetal bovine serum (FBS, Helena Bioscience, Sunderland, UK) and kept at 37˚C in a humidified atmosphere of air containing 5% $CO_2$. Cells were maintained in the logarithmic phase of growth by passaging 2–3 times weekly. Staining of methanol/acetone fixed cells with Hoescht 33258 was used to confirm that cell lines were free from mycoplasma.

Hypoxic (5% $CO_2$, 0.1% $O_2$, 94.9% $N_2$) conditions were generated in a hypoxic incubator (Cell House 170, Heto-Holten (UK) Ltd, Surrey, UK).

### Flow cytometry

Antigen expression was confirmed via flow cytometry. In brief, following a 1 hour incubation with 3% fetal bovine serum in phosphate-buffered saline (FBS-PBS) to block non-specific antibody binding, cells were incubated with the relevant primary antibody (EGFR1, or DH8.3) for 1 hour, followed by fluorescein-conjugated F(ab')2 rabbit anti-mouse immunoglobulins (1:20 dilution; DAKO, High Wycombe, UK). Binding of primary antibody was analysed using a Becton Dickenson FACS machine.

### Growth curves

#### *In vitro*

DH-EGFR-E and DH-EΔ801-P cells were plated in triplicate at the same starting concentration, in media containing 10% FBS. Cells were harvested on days one to five of culture, and counted on an automated particle and size analyser (Coulter Z-2, Coulter Electronics Ltd, Luton, UK).

#### *In vivo*

Using a previously established method (Hills et al, 1995), tumours were established in BALB/c nu/nu mice by subcutaneous injection into the flank of $5 \times 10^6$ cells of each of the cell lines described above. Tumours were measured in the longest axis (L) and the axis at 90˚ to the longest axis (W) by slide caliper 2–3 times a week. Tumour volume (TV) in cubic millimetres was calculated by the formula volume = $(L \times W^2)/2$ (Inaba et al, 1989) and the mean tumour volume (± standard deviation) for each treatment group calculated and graphed.

### Measurement of VEGF protein levels

Cells were plated at a density of $2 \times 10^5$ cells 2 ml media$^{-1}$ well$^{-1}$ in 6-well culture plates (Falcon, Becton Dickinson Labware, NJ, USA) and allowed to grow to desired confluence. At this time the culture medium was aspirated and replaced with fresh assay medium with or without inhibitors of PI3 kinase, IGF-1, EGF or blocking antibody (DH8.3). Following 16 hours hypoxia (0.1% $O_2$) or normoxia, medium was collected, cellular debris removed by centrifugation, and medium stored at –20˚C until VEGF quantification. Levels of VEGF in conditioned medium were quantified using a commercially available mouse VEGF ELISA kit (R&D Systems, Minneapolis, MN). Results between wells were standardized according to the cell number per well as measured using an automated (Coulter) cell counter.

### Effect of confluence and % FBS on VEGF secretion

Cells were plated in triplicate in 6-well plates at increasing cell densities. When the wells with the highest number of cells reached 90% confluence, plates were processed for VEGF in the usual manner. At the end of normoxic or hypoxic incubation cells were removed with PBS/EDTA and cell count determined via a Coulter counter.

To assess the effect of FBS, cells were grown to 70% confluence in medium containing 10% FBS, then medium changed to 0, 1 or 10% FBS for 16 hours hypoxia or normoxia. Supernatant was processed as above.

### Effect of anti-EGFRvIII on xenograft growth

Commencing on day 0 (D0), where D0 is the day of cell pellet inoculation, mice were treated with 1 mg per mouse of DH8.3 antibody every other day for a total of 4 injections. Antibody was administered intravenously through the tail vein. The frequency of dosing was based on the known plasma half life of DH8.3 in vivo (*t* 1/2 β 66 hours) (Hills et al, 1995). Control mice received PBS alone in the same schedule. Mice were measured and tumour volume calculated as described previously.

At the completion of the study, xenografts were resected and frozen for further analysis. VEGF levels in tumour sections were determined via VEGF ELISA, following homogenization of samples in a buffer containing 20 mM HEPES, 1.5 mM EDTA, 0.5 mM benzamidine, 0.5 mM phenylmethylsulfonylfluoride and 10 μg $ml^{-1}$ ovomucoid trypsin inhibitor (Sacks et al, 1993) and loading of a standardized amount of protein into each well.

### Statistics

All experiments were carried out in triplicate and repeated at least twice. Statistical analysis was carried out with Sigmastat for Windows (Jandel Scientific, San Rafael, CA, USA). Comparisons between groups were made using the unpaired *t*-test.

### RESULTS

### Expression of EGFR type III

Expression of full length or truncated EGFRvIII was confirmed via flow cytometry (Figure 1). DH-EΔ801-P was shown to express EGFRvIII, and the empty vector control expressed neither mutant nor full length receptor. DH-EGFR-E expressed full length, but not mutant EGFR. The levels in DH-EΔ801-P are similar to those expressed in glioblastomas with gene amplification (Hills et al, 1995).

### In vitro and in vivo growth

No significant difference in growth rates in vitro were seen between cells expressing full length or truncated EGFR when grown in medium containing 10% FBS. However in vivo there were dramatic differences between the growth rates, when cell lines expressing the mutant receptor growing rapidly to form large tumours (Figure 2).

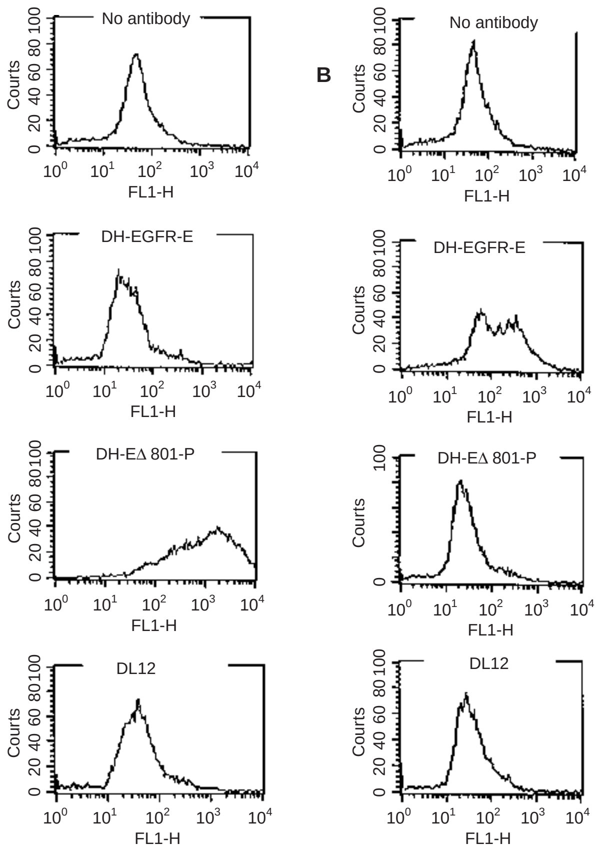

**Figure 1** Flow cytometry of EGFR expression in cell lines. Presence of EGFRvIII was assessed by the ability to bind DH8.3 (anti-EGFR specific antibody) (**A**), and full length EGFR by binding to EGFR1 (**B**)

### Increased VEGF secretion in mutant cells following hypoxia

Using cells subconfluent at the beginning of hypoxic incubation, VEGF secretion by cells expressing mutant EGFR receptor (DH-EΔ801-P) was consistently upregulated following exposure to 0.1% hypoxia for the duration of 16 hours (Figure 3). The fold induction following hypoxia was variable between experiments (range 3.5–6.6), which may be attributable to minor differences in confluence (see below) but always achieved statistical significance. In contrast cells expressing full length EGFR showed less than 2-fold increase in VEGF secretion between normoxia and hypoxia. Baseline (normoxic) levels of VEGF in full length EGFR expressing cells was similar or slightly elevated compared to mutant cells.

### Cell confluence and serum starvation can effect VEGF levels

In cells expressing the mutant receptor, VEGF secretion into the supernatant following hypoxic stress increased with confluence, peaking at 60–70% confluence. Of note hypoxic VEGF levels were higher at subconfluence (mean ±SD, 2112 pg $10^6$ $cells^{-1}$ $ml^{-1}$ ± 215) than confluence (1113 pg $10^6$ $cells^{-1}$ $ml^{-1}$ ± 112) ($P$ = 0.002). Normoxic VEGF levels did not differ significantly with degree of confluence. There was a minor effect of percentage of FBS in the medium on VEGF secretion. Again differences were

 *© 2001 Cancer Research Campaign*

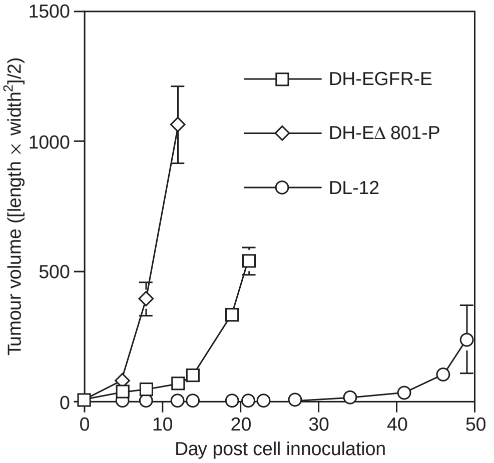

**Figure 2** In vivo growth of cell lines. $5 \times 10^6$ cells were innoculated into the flank of BALB/c nu/nu mice and tumours measured 2–3 times a week. Mutant cells formed tumours rapidly over a 7–10 day period. Each curve shown represents mean ± SD of 7 mice

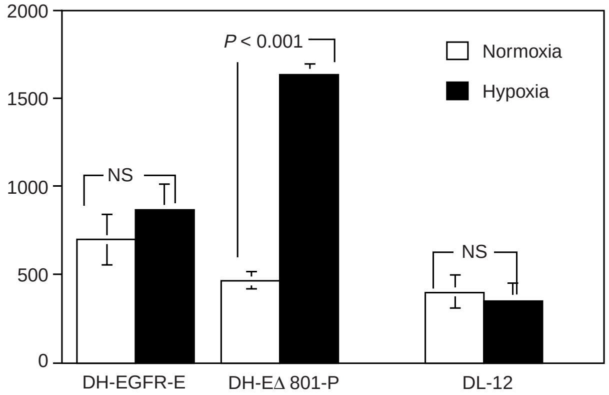

**Figure 3** Comparison of VEGF levels in the supernatant of cells expressing full length EGFR (DH-EGFR-E), mutant receptor (DH-EΔ801-P) and empty vector control (DL-12). Levels of VEGF in the cell supernatant after 16 hours of normoxic or hypoxic incubation were quantified by ELISA assay standardized for cell number. Results shown represent mean ± SD of triplicate samples. NS: not significant only significant following hypoxia incubation. Hypoxic VEGF levels of cells exposed to 0% FBS (1684 pg $10^6$ cells$^{-1}$ ml$^{-1}$ ± 48) were significantly higher than cells grown in 10% FBS (1397 pg $10^6$ cells$^{-1}$ ml$^{-1}$ ± 63.5) ($P = 0.003$).

### In vitro and in vivo effects of anti-EGFR antibody

In vitro, 10 μg ml$^{-1}$ of EGFRvIII specific monoclonal antibody DH8.3 was added to fresh culture medium immediately before hypoxic incubation. Reduction in hypoxic VEGF levels was seen in cells expressing mutant EGFR ($P = 0.003$), but not for cells with full length receptor ($P = 0.07$). In vivo, there was suppression of growth of DH-EΔ801P (mutant EGFR) xenografts when antibody was administered intravenously for 4 doses at 48-hour intervals, for the duration of antibody administration. This was reversible once antibody was discontinued (Figure 4).

VEGF levels in tumours in antibody-treated mice showed lower levels than in the controls. The antibody treated group ($n = 7$), range was 33–175 pg mg$^{-1}$ cytosol protein, median 72 pg mg$^{-1}$ and

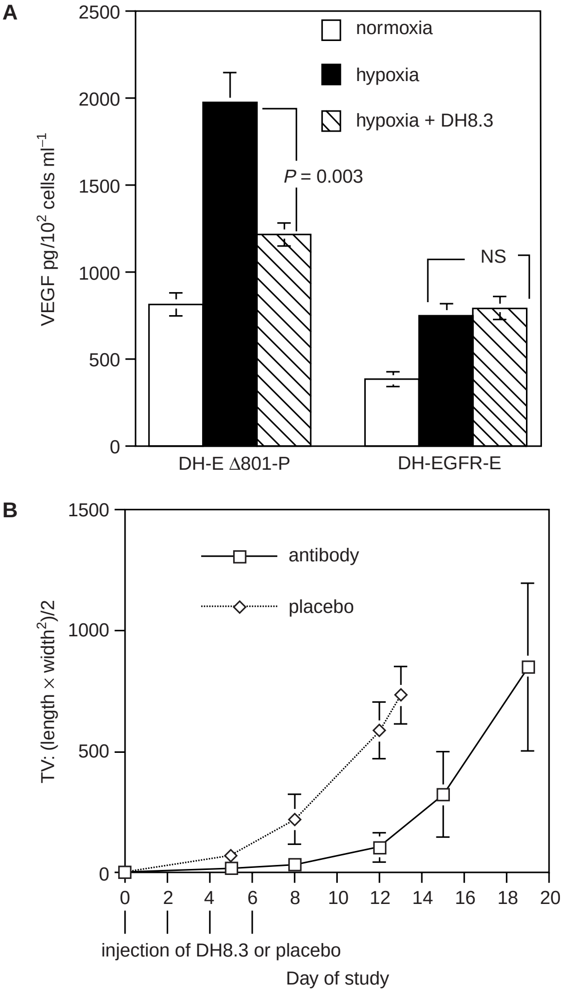

**Figure 4** Effect of administration of anti-EGFRvIII antibody (DH8.3) in vitro and in vivo. In vitro (**A**) 10 μg ml$^{-1}$ of antibody was added to culture medium of mutant (DH-EΔ801-P) or wild-type (DH-EGFR-E) cells immediately prior to 16 hours of 0.1% hypoxia, and VEGF secretion compared to normoxic and hypoxic cells without antibody. In vivo (**B**) 1 mg mouse$^{-1}$ of DH8.3 or placebo (PBS alone) was administered via tail vein injection on alternate days for 4 doses, commencing on the day of cell pellet inoculation (Day 0). The mean tumour volume ± SD of 7 mice in each treatment group are shown. NS: not significant

SD 45 pg mg$^{-1}$, whereas in the controls ($n = 7$), the range was 83–220 pg mg$^{-1}$ cytosol protein, median 110 pg mg$^{-1}$, SD 49 pg mg$^{-1}$ ($P = 0.08$, Mann–Whitney Rank test).

### Mechanism of hypoxic induction of VEGF

The response to EGF was assessed, and as anticipated there was no evidence of stimulation of VEGF production by EGFR in mutant cell lines. However, when added to the culture medium of wild-type receptor cells there was a significant increase in VEGF production in EGF-stimulated, compared with unstimulated, cells ($P = 0.01$). This increase was only seen in cells incubated under normoxic conditions with no further increase following hypoxia (Figure 5).

Inhibitors of PI3 kinase (wortmannin (5 μm) or LY 294002 (20 μm)) were added to fresh serum-free media immediately prior to 16 hours normoxia or hypoxia. Following hypoxia, induced levels of VEGF were inhibited in DH-EΔ801-P cells compared to cells exposed to hypoxia alone by 63 to 80%. P13 kinase inhibitors did

© 2001 Cancer Research Campaign

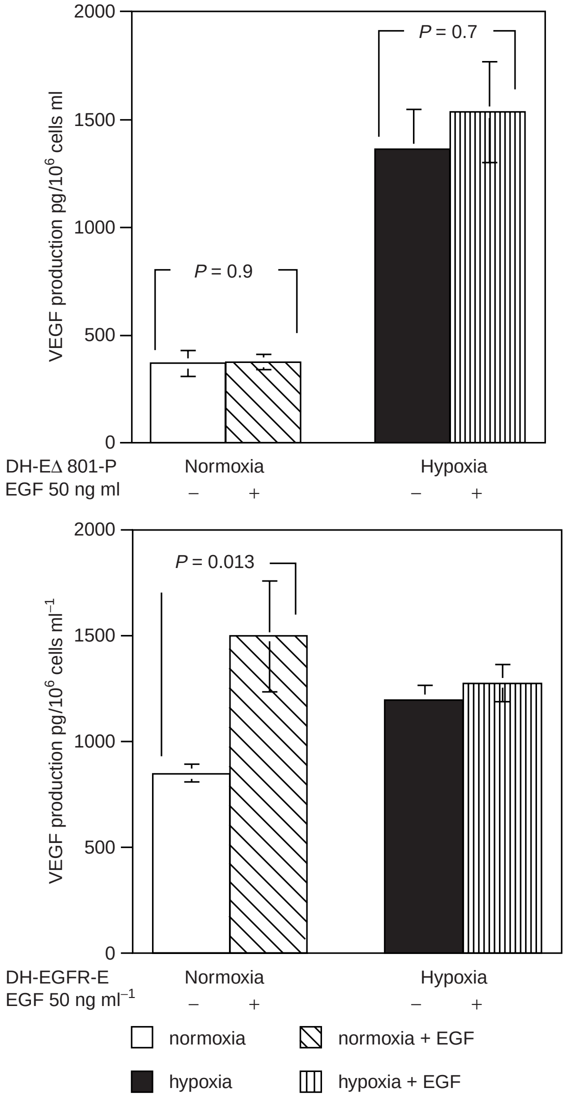

**Figure 5** Effect of addition of exogenous EGF (50 ng ml$^{-1}$) to cells expressing full length (DH-EGFR-E) or mutant (DH-EΔ801-P) receptor. Supernatant was collected after 16 hours incubation under normoxic or hypoxic (0.1% oxygen) conditions in serum-free medium, and VEGF levels assayed. Mean ± SD of triplicate results are shown

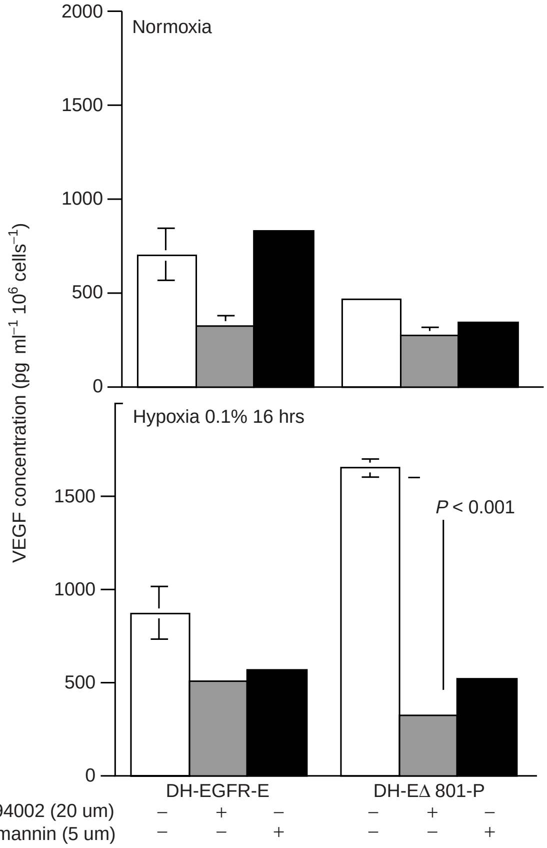

**Figure 6** Effects of P13 kinase inhibitor LY 294002 on VEGF induction by hypoxia. LY 294002 was added to fresh serum-free medium of cells expressing high (DH-EΔ801-P) levels of mutant receptor, or wild-type cells (DH-EGFR-E) immediately prior to exposure to 16 hours normoxia (**A**) or 0.1% hypoxia (**B**). At the end of this incubation supernatant was collected and VEGF quantified. VEGF mean ± SD of triplicate samples are shown not suppress VEGF levels to below baseline (normoxic) values in any cell line studied (Figure 6). Increasing the dose of LY 294002 (40 µm) failed to produce a larger reduction in VEGF levels to below baseline levels (results not shown).

IGF-1 was added to subconfluent cells cultured in serum-free conditions for 8 hours prior to the addition of IGF-1 to minimize the effects of other growth factors in culture medium. The dose of IGF-1 chosen (100 ng ml$^{-1}$) was based on published studies demonstrating 50–100 ng ml$^{-1}$ of IGF-1 saturated VEGF mRNA and protein production in normoxic colo 205 cells (Warren et al, 1996). VEGF secretion in DH-EΔ801-P cells grown under normoxic conditions increased following addition of IGF-1 to levels not significantly different from hypoxic values ($P$ = 0.5). Addition of IGF-1 to cells exposed to 16 hours hypoxia produced no significant change in VEGF levels ($P$ = 0.2) (Figure 7), suggesting that the system was already maximally stimulated. In empty vector control lines, IGF-1 did not increase VEGF secretion under normoxic ($P$ = 0.9) or hypoxic ($P$ = 0.4) conditions. Preincubation of cells with LY 294002 prior to addition of IGF-1, partially inhibited the increase in VEGF secretion seen following 16 hours of hypoxia (data not shown).

### DISCUSSION

EGFR is a transmembrane glycoprotein with an external binding domain and an intracellular tyrosine kinase domain. Following ligand binding, EGFR dimerizes and is autophosphorylated on several tyrosine residues in the intracellular domain and initiates a kinase cascade that activates members of the MAP kinase pathway (Lacal and Carnero, 1994). In EGFRvIII, it is known that the loss of exons 2–7 in the extracellular domain leads to the receptor being constitutively active (Ekstrand et al, 1995). Cell lines established from tumours that express endogenous EGFRvIII lose expression of the variant receptor in vitro in tissue culture (Humphrey et al, 1990). Increased tumorigenicity of human glioblastoma cell line (U87) xenografts bearing a mutant plus full length EGFR, compared to full length receptor alone, has been previously shown (Niskikawa et al, 1994). Both observations suggest a growth disadvantage for mutant cells in vitro, and/or selection for mutant cells in vivo. We describe here enhanced tumorigenicity in vivo of NIH3T3 cells transfected with mutant EGFR as compared to full length EGFR, which occurred in the

*© 2001 Cancer Research Campaign*

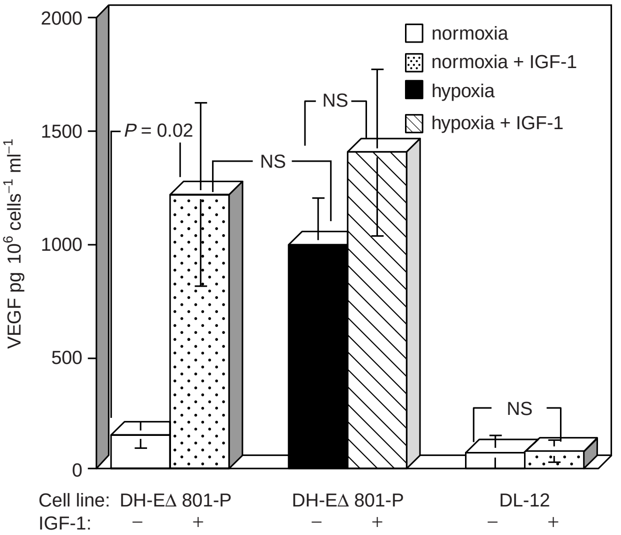

**Figure 7** Effects of IGF-1 on VEGF secretion. Subconfluent cells expressing the mutant EGFR receptor (DH-EΔ801-P) or empty vector control (DL-12) were placed in serum-free medium for 8 hours prior to addition of IGF-1 100 ng $ml^{-1}$ and incubation for 16 hours in hypoxia or normoxia. Supernatant was collected and VEGF quantified via ELISA assay. Mean ± SD of triplicate results are shown absence of any growth advantage in vitro. This provides a suitable model for investigation of stimuli responsible for enhanced in vivo growth.

VEGF expression has been demonstrated in both malignant and non-malignant cells, but is over-expressed in many malignant cells (Takahashi et al, 1995). Stimulation of glioma cells expressing full length EGFR by EGF resulted in a 25–125% increase in VEGF secretion under normoxic conditions (Goldman et al, 1993). We also demonstrated an increase in VEGF in EGF-stimulated wild-type EGFR cells, but not in cells expressing mutant receptor. Studies of VEGF distribution in tumours have shown localization to necrotic and ischaemic areas, suggesting an interaction between hypoxia and induction of VEGF (Minchenko et al, 1994). In vitro, the relationship between VEGF and hypoxia has been demonstrated in many cell lines. Given that EGFRvIII is expressed on a high percentage of glioblastoma cells and glioblastoma multiforme is a highly anaplastic tumour with abundant proliferating capillaries and paradoxically areas of necrosis, we investigated the relationship between hypoxia, VEGF secretion, and expression of mutant or full length EGFR. In the absence of exogenous EGF, full length EGFR cells exposed to hypoxia produced only a minor increase in VEGF compared to significant induction in mutant cells. Hypoxia may therefore be a driving force for VEGF gene expression in these cells.

Expression of EGFvIII, compared to full length receptor, is associated with lower levels of ERK (Antonyak et al, 1998), higher levels of JNK activity, and constitutively active Pl3 kinase (Moscatello et al, 1998). Pl3 kinase may be responsible for the high levels of JNK activity and contribute to the transformed phenotype in that inhibition of Pl3 kinase activity results in down-regulation of JNK activity and correlated with loss of transformed morphology (Moscatello et al, 1998). Kinase activity of wild-type and mutated EGFR can be differentially blocked by various tyrosine kinase inhibitors, consistent with the idea that the conformational changes induced by ectodomain deletion in EGFRvIII differs from ligand activated wild-type EGFR (Han et al, 1996).

Pl3 kinase is involved in signal transduction that is activated by ras and other dominant oncogenes and growth factors, and may play a role in regulating the angiogenic switch (Arbieser et al, 1997). Serine/threonine kinase Akt/protein kinase B (PKB) is a major target of Pl3 kinase-mediated signals (Gerber et al, 1998). Akt/PKB is rapidly activated by insulin and a number of growth factors including platelet-derived growth factor, EGF and IGF-1 (Franke et al, 1997; Holst et al, 1998). Using both wortmannin and LY294002, reduction in VEGF protein secretion was seen in mutant cells in response to hypoxia but not basal levels, suggesting that Pl3 kinase has a major role in the hypoxic response, but constitutive activation alone was insufficient. Giaccia showed transfection of Pl3 kinase enhanced hypoxic induction of a reporter construct with hypoxia inducible factor-1 alpha site, although the mechanism is unknown (Mazure et al, 1997).

A recent study showed a role for Pl3 kinase in regulation of VEGF by the wild-type and truncation mutant EGFr (Maity et al, 2000). However in that study Pl3 kinase inhibition down-regulated basal VEGF but had no effect on hypoxia-inducible VEGF, in contrast to our study.

VEGF expression is regulated by numerous cytokines and growth factors including interleukins 1 and 6, transforming growth factor beta, and basic fibroblast growth factor, the effect of which is dependent on the tumour system studied (Akagi et al, 1998). IGF-1, a homologue of insulin, and its receptor contribute to neoplastic transformation and the development of tumorigenicity through autocrine and paracrine mechanisms (Baserga, 1995; Warren et al, 1996) and is an important mitogen for many tumour types. It shares with hypoxia and insulin the ability to induce expression of genes encoding for the glucose transporters (Glut 1 and 3), erythropoietin and VEGF (Zelzer et al, 1998). Basal levels of VEGF, whilst varying between cell lines, have been shown to increase following stimulation with IGF-1 in cancer cells and non-malignant cell lines (Singh and Rubin, 1993; Warren et al, 1996; Akagi et al, 1998). This response has been reported in lines in which it induced a proliferative response and in lines in which it did not, and therefore appears to be distinct from any effects on cell proliferation (Zelzer et al, 1998). In transformed NIH3T3 cells expressing mutant EGF receptor, IGF-1 increased the secretion of VEGF levels under normoxic conditions to levels equivalent to hypoxic levels. There was no significant further increase in hypoxic cells stimulated with IGF-1, suggesting both operated via a final common pathway. This pathway was also inhibited by Pl3 kinase inhibitors. These data show that mutant EGFR is associated with enhanced signalling by Pl3 kinase that synergizes with hypoxia or IGF-1 under normoxic conditions to regulate VEGF.

In vitro blocking antibodies inhibited hypoxic induction of VEGF suggesting that constitutive activation was reversed or the mutant receptor down-regulated. In vivo this correlated with reduced tumour growth. Median VEGF levels were also lower in the treated group as were the lowest and highest VEGF levels compared to the controls, although this did not reach statistical significance. The mechanism of enhanced tumorigenicity in vivo of mutant EGFRvIII cells does not appear to be directly due to an effect on increased cell growth in that when removed from exogenous stimuli in vitro, no differences in growth are observed, but rather reflects interactions between environmental stimuli and cells bearing mutant receptor. Tumour microenvironment is likely to play a critical role in regulating cell viability. Regions of low oxygen tension are common to solid tumours and appear to signal an increase in angiogenic gene expression and increase apoptosis.

*© 2001 Cancer Research Campaign*

The in vitro results provide a potential explanation for the in vivo growth differences, in that hypoxia could synergize with mutant EGFR, or in non-hypoxic areas with paracrine growth factors to enhance VEGF production.

Mutant EGFr may also interact with other oncogenes, particularly H-ras to induce VEGF. Although in our study there was no effect of mutant EGFr on basal VEGF levels, in a cell line with high Ras activity there was an increase in normoxic VEGF levels (Feldkamp et al, 1999). The increase induction of VEGF by hypoxia was much less than in our experiments, 25% greater than the parent cell line. Our study shows the differing effects in a low versus the high Ras background.

Mutated EGFR thus provides a potential target for tumour-specific therapy, although antibodies against wild-type receptors have also shown selective effects against tumours (Mendelsohn, 1998). The results presented here show that one mechanism by which oncogenes may function is enhancement of response to hypoxia, without change in basal VEGF expression, in contrast to previous results with wild-type EGF receptor. It may be that the splice variant is specifically selected by the hypoxic micro-environment as are mutations of p53 (Graeber et al, 1996). We have previously shown that the hypoxic stress pathway regulated by hypoxic inducible factor-1 alpha is critical for in vivo growth of tumours (Maxwell et al, 1997) and interactions with mutant EGFR may provide a molecular mechanism for amplifying this hypoxic switch. This study supports both the use of anti-EGFR antibodies and Pl3 kinase inhibitors to block in vivo tumour growth mediated by mutant EGFR.

### REFERENCES

Akagi Y, Liu W, Zebrowski B, Xie K and Ellis LM (1998) Regulation of vascular endothelial growth factor expression in human colon cancer by insulin-like growth factor-1. *Cancer Res* **58**: 4008–4014

Antonyak MA, Moscatello DK and Wong AJ (1998) Constitutive activation of c-Jun N-terminal kinase by a mutant epidermal growth factor receptor. *J Biol Chem* **273**: 2817–2822

Arbieser JL, Moses MA, Fernandez CA, Ghiso N, Cao Y, Klauber N, Frank D, Brownlee M, Flynn E, Parangi S and Byers R (1997) Oncogenic H-ras stimulates tumor angiogenesis by two distinct pathways. *Proc Natl Acad Sci USA* **94**: 861–866

Baserga R (1995) The insulin-like growth factor I receptor: a key to tumor growth? *Cancer Res* **55**: 249–252

De Jong JS, Van Diest PJ, Van der Valk P and Baak JPA (1998) Expression of growth factors, growth-inhibiting factors, and their receptors in invasive breast cancer. II: correlation with proliferation and angiogenesis. *J Pathol* **184**: 53–57

Ekstrand AJ, Sugawa N, James CD and Collins VP (1992) Amplified and rearranged epidermal growth factor receptor genes in human glioblastomas reveal deletions of sequences encoding portions of the N-and/or C-terminal tails. *Proc Natl Acad Sci USA* **89**: 4309–4313

Ekstrand AJ, Longo N, Hamid ML, Olsen JJ, Liu L, Collins VP and James CD (1994) Functional characterization of an EGFR receptor with a truncated extracellular domain expressed in glioblastomas with EGF receptor gene amplification. *Oncogene* **9**: 2312–2320

Ekstrand AJ, Liu L, He J, Hamid ML, Longo N, Collins VP and James CD (1995) Altered subcellular location of an activated and tumor – associated epidermal growth factor receptor. *Oncogene* **10**: 1455–1460

Feldkamp MM, Lau N, Rak J, Kerbel RS and Guha A (1999) Normoxic and hypoxic regulation of vascular endothelial growth factor (VEGF) by astrocytoma cells is mediated by Ras. *Int J Cancer* **18**: 118–124

Fidler IJ and Ellis LM (1994) The implications of angiogenesis to the biology and therapy of cancer metastasis. *Cell* **79**: 185–188

Folkman J (1990) What is the evidence that tumours are angiogenesis dependent? *J Natl Cancer Inst* **80**: 4–6

Franke TF, Kaplan DR and Cantley LC (1997) P13K: downstream AKTion blocks apoptosis. *Cell* **88**: 435–437

Gerber H-P, McMurtrey A, Kowalski J, Yan M, Keyt BA, Dixit V and Ferrara N (1998) Vascular Endothelial growth factor regulates endothelial cell survival through the phosphatidylinositol 3′kinase/Akt signal transduction pathway. *J Biol Chem* **273**: 30336–30343

Goldman CK, Kim J, Wong W-L, King V, Brock T and Gillespie GY (1993) Epidermal growth factor stimulates vascular endothelial growth factor production by human malignant glioma cells: A model of glioblastoma multiforme pathophysiology. *Mol Biol Cell* **4**: 121–133

Graeber TG, Osmanian C, Jacks T, Housman DE, Koch CJ, Lowe SW and Giaccia AJ (1996) Hypoxia-mediated selection of cells with diminished apoptotic potential in solid tumours. *Nature* **379**: 88–91

Guha A, Feldkamp MM, Lau N, Boss G and Pawson T (1997) Proliferation of human malignant astrocytomas is dependent on Ras activation *Oncogene* **15**: 2755–2766

Gullick WJ (1991) Prevalence of aberrant expression of the epidermal growth factor receptor in human cancers. *Brit Med Bull* **47**: 87–98

Han Y, Caday CG, Nanda A, Cavenee WK and Huang HJ (1996) Tyrphostin AG 1478 preferentially inhibits human glioma cells expressing truncated rather than wild-type epidermal growth factor receptors. *Cancer Res* **56**: 3859–3861

Harris AL, Nicholson S, Sainsbury R, Wright C and Farndon J (1992) Epidermal growth factor receptor and other oncogenes as prognostic markers. *Natl Cancer Inst Monogr* **11**: 181–187

Hills D, Rowlinson-Busza G and Gullick WJ (1995) Specific targeting of a mutant, activated EGF receptor found in glioblastoma using a monoclonal antibody. *Int J Cancer* **63**: 537–543

Holst LS, Mulder H, Manganiello V, Sundler F, Ahren B, Holm C and Degerman E (1998) Protein kinase B is expressed in pancreatic beta cells and activated upon stimulation with insulin-like growth factor I. *Biochem Biophys Res Commun* **250**: 181–186

Humphrey PA, Wong AJ, Vogelstein B, Zalutsky MR, Fuller GN, Archer GE, Friedman HS, Kwatra MM, Bigner SH and Bigner DD (1990) Anti-synthetic peptide antibody reacting at the fusion junction of deletion-mutant epidermal growth factor receptors in human glioblastoma. *Proc Natl Acad Sci USA* **87**: 4207–4211

Inaba M, Kobayashi T, Tashiro T, Sakurai Y, Ohnishi Y, Ueyama Y and Nomura T (1989) Evaluation of anti tumour activity in a human breast tumour / nude mouse model with a special emphasis on treatment dose. *Cancer* **64**: 1577–1582

Lacal JC and Carnero A (1994) Regulation of ras proteins and their involvement in signal transduction pathways. *Oncol Rep* **1**: 677–693

Levy AP, Levy NS, Wegner S and Goldberg MA (1995) Transcriptional regulation of the rat vascular endothelial growth factor gene by hypoxia. *J Biol Chem* **270**: 13333–13340

Maity A, Pore N, Lee J, Solomon D and Orourke DM (2000) Epidermal growth factor receptor transcriptionally up-regulates vascular endothelial growth factor expression in human glioblastoma cells via a pathway involving phosphatidylinositol 3′-kinase and distinct from that induced by hypoxia. *Cancer Res* **60**: 5879–5886

Maxwell PH, Dachs GU, Gleade JM, Nicholls LG, Harris AL, Stratford IJ, Hankinson O, Pugh CW and Ratcliffe PJ (1997) Hypoxia-inducible factor-1 modulates gene expression in solid tumours and influences both angiogenesis and tumour growth. *Proc Natl Acad Sci USA* **94**: 8104–8109

Mazure NM, Chen EY, Laderoute KB and Giaccia AJ (1997) Induction of vascular endothelial growth factor by hypoxia is modulated by a phosphatidylinositol 3-kinase/Akt signaling pathway in Ha-ras-transformed cells through a hypoxia inducible factor-1 transcriptional element. *Blood* **90**: 3322–3331

Mendelsohn J (1998) Epidermal growth factor receptor inhibition by a monoclonal antibody as anticancer therapy. *Clin Cancer Res* **3**: 2703–2707

Minchenko A, Bauer T, Salceda S and Caro J (1994) Hypoxic stimulation of vascular endothelial growth factor expression in vitro and in vivo. *Lab Invest* **71**: 374–379

Moscatello DK, Holgado-Madruga M, Godwin AK, Ramirez G, Gunn G, Zoltick PW, Biegel JA, Hayes RL and Wong AJ (1995) Frequent expression of a mutant epidermal growth factor receptor in multiple human tumors. *Cancer Res* **55**: 5536–5539

Moscatello DK, Holgado-Madruga M, Emlet DR, Montgomery RB and Wong AJ (1998) Constitutive activation of phosphatidylinositol 3-kinase by a naturally occuring mutant epidermal growth factor receptor. *J Biol Chem* **273**: 200–206

Nishikawa R, Ji X.-D, Harmon RC, Lazar CS, Gill GN, Cavenee WK and Huang H.-JS (1994) A mutant epidermal growth factor receptor common in human glioma confers enhanced tumorigenicity. *Proc Natl Acad Sci USA* **91**: 7727–7731

Petit AM, Rak J, Hung MC, Rockwell P, Goldstein N, Fendly B and Kerbel RS (1997) Neutralizing antibodies against epidermal growth factor and

*© 2001 Cancer Research Campaign*

ErbB-2/neu receptor tyrosine kinases down-regulate vascular endothelial growth factor production by tumour cells in vitro and in vivo: angiogenic implications for signal transduction therapy of solid tumors. *Am J Pathol* **151**: 1523–1530

Rak J, Mitsuhashi Y, Bayko J, Filmus J, Shirasawa S, Sasazuki T and Kerbel RS (1995) Mutant *ras* oncogenes upregulate VEGF/VPF expression: Implications for induction and inhibition of tumour angiogenesis. *Cancer Res* **55**: 4575–4580

Sacks NPM, Smith K, Norman AP, Greenail M, LeJeune S and Harris AL (1993) Cathepsin D levels in primary breast cancers: relationship with epidermal growth factor receptor, oestrogen receptor and axillary nodal status. *Eur J Cancer* **29**: 426–428

Salomon DS, Brandt R, Ciardiello F and Normanno N (1995) Epidermal growth factor-related peptides and their receptors in human malignancies. *Crit Rev Oncol Hematol* **19**: 183–232

Schmidt M, Reiser P, Hills D, Gullick WJ and Wels W (1998) Expression of an oncogenic mutant EGF receptor markedly increases the sensitivity of cells to an EGFR-receptor-specific antibody toxin. *Int J Cancer* **75**: 878–884

Scott PAE, Gleadle JM, Bicknell R and Harris AL (1998) Role of the hypoxia sensing system, acidity and reproductive hormones in the variability of vascular endothelial growth factor induction in human breast cancer cell lines. *Int J Cancer* **75**: 706–712

Shweiki D, Itin A, Soffer D and Keshet E (1992) Vascular endothelial growth factor induced by hypoxia may mediate hypoxia-initiated angiogenesis. *Nature* **359**: 843–845

Singh P and Rubin N (1993) Insulin like growth factors and binding proteins in colon cancer. *Gastroenterology* **105**: 1218–1237

Takahashi Y, Kitadai Y, Bucana CD, Cleary KR and Ellis CM (1995) Expression of vascular endothelial growth factor and its receptor. KDR correlates with vascularity, metastasis, and proliferation of human colon cancer. *Cancer Res* **55**: 3964–3968

Warren RS, Yuan H, Matli MR, Ferrara N and Donner DB (1996) Induction of vascular endothelial growth factor by insulin-like growth factor 1 in colorectal carcinoma. *J Biol Chem* **271**: 29483–29488

Waterfield MD, Mayes ELV, Stroobant P, Bennet PLP, Young S, Goodfellow PN, Banting GS and Ozanne B (1982) A monoclonal antibody to the human epidermal growth factor receptor. *J Cell Biochem* **20**: 149–161

Whitmarsh AJ and Davis RJ (1996) Transcription factor AP-1 regulation by mitogen-activated protein kinase signal transduction pathways. *J Mol Med* **74**: 589–607

Wikstrand CJ, Hale LP, Batra SK, Hill ML, Humphrey PA, Kurpad SN, McLendon RE, Moscatello D, Pegram CN, Reist CJ, Traweek ST, Wong AJ, Zalutsky MR and Bigner DD (1995) Monoclonal antibodies against EGFRvIII are tumour specific and react with breast and lung carcinomas and malignant gliomas. *Cancer Res* **55**: 3140–3148

Wong AJ, Ruppert JM, Bigner SH, Grzteschik CH, Humphrey PA, Bigner DS and Vogelstein B (1992) Structural alterations of the epidermal growth-factor receptor gene in human gliomas. *Proc Natl Acad Sci USA* **89**: 2965–2969

Zelzer E, Levy Y, Kahana C, Shilo B-Z, Rubinstein M and Cohen B (1998) Insulin induces transcription of target genes through the hypoxia-inducible factor HIF-1α/ARNT. *EMBO J* **17**: 5085–5094

© 2001 Cancer Research Campaign 
